# p53 increase mitochondrial copy number via up-regulation of mitochondrial transcription factor A in colorectal cancer

**DOI:** 10.18632/oncotarget.12514

**Published:** 2016-10-07

**Authors:** Shilei Wen, Jinhang Gao, Linhao Zhang, Hongying Zhou, Dingzhi Fang, Shi Feng

**Affiliations:** ^1^ Department of Human Anatomy, School of Preclinical and Forensic Medicine, West China Medicine College, Sichuan University, Chengdu 610041, China; ^2^ Division of Peptides Related with Human Diseases, State Key Laboratory of Biotherapy, West China Hospital, Sichuan University, Chengdu 610041, China; ^3^ Department of Gastroenterology, West China Hospital, Sichuan University, Chengdu 610041, China; ^4^ Department of Biochemistry and Molecular Biology, School of Preclinical and Forensic Medicine, West China Medicine College, Sichuan University, Chengdu 610041, China

**Keywords:** colorectal cancer, p53, mitochondrial transcription factor A, mitochondrial DNA copy number

## Abstract

In colorectal cancer, no study has been carried out discovering the relationship among p53, mitochondrial transcription factor A (TFAM) expression and change of mitochondrial DNA (mtDNA) copy number. In our study, co-expression of p53 and TFAM was observed in colon adenocarcinoma tissues, paracancerous tissues and 9 colorectal cancer cell lines. Then, a significant linear correlation was established between either p53 or TFAM expression and advanced TNM stage, positive lymph nodes and low 5-year survival rate in patients with colon adenocarcinoma. Additionally, advanced TNM stage, large tumor burden, presence of distant metastasis, and high TFAM expression were significantly related to poor overall 5-years survival. Moreover, alteration of p53 expression could change TFAM expression but TFAM could not influence p53 expression, and p53 could enhance TFAM expression via binding to TFAM promoter. While, both of p53 and TFAM expression could incrase mtDNA copy number *in vitro*. In conclusions, p53 might incrase mtDNA copy number through its regulation on TFAM expression via TFAMpromoter.

## INTRODUCTION

Colorectal cancer, accounting for 8% of newly diagnosed cancer, is the third leading type of cancer which causes the death of both men and women [1]. However, the mechanism of how colorectal cancer develops is still elusive. In order to provide novel treatment to colorectal cancer, efforts on understanding its progression should be made.

mtDNA is the DNA inside of mitochondria, which functions to encode polypeptides of the oxidative phosphorylation complexes [2]. Given its role in metabolism, it is not surprising that alteration of mtDNA is also related to human diseases, including cancer [3]. Our previous work found that mtDNA copy number was higher in cancerous tissues than in corresponding paracancerous tissues in patients with colorectal cancer [4]. Various mechanisms are responsible for the regulation of mtDNA copy number [5–7]. Among them, mitochondrial transcription factor A (TFAM) is considered essential because it could maintain the structure and stimulate the transcription of mtDNA [8]. However, though it has been observed that TFAM is associated with the prognosis of patients with colorectal cancer [9], not much has been done to investigate whether there is a role of TFAM in the development of colorectal cancer.

p53 gene is a tumor suppressor gene encoding p53 protein, whose primary function is to arrest cell-cycle and cause apoptosis when necessary [10]. Mutation of p53 gene is found in approximately 35% to 55% of the patients with colorectal cancer [11], which promotes oncogenesis [12]. It has long been observed an accumulation of p53 protein in cancer [13], though this overexpression is not necessarily associated with p53 gene mutation [14, 15]. In addition, p53 was announced to be a regulator of metabolism recently [16], and some report has been published, describing p53 regulation on mtDNA [17]. Thus, it is interesting to explore whether p53 overexpression could influence mtDNA copy number in colorectal cancer.

Our research aims to reveal p53 and TFAM expression and their correlation with clinicopathological characteristics of patients with colon adenocarcinoma, the regulative relationship between p53 and TFAM, and whether p53 and TFAM could regulate mtDNA copy number in colorectal cancer cell lines.

## RESULTS

### Patient characteristics

As shown in Table 1, the clinical factors of the 90 patients with colon cancer were recorded. Among the patients, 52 (57.8%) were men and the remaining 38 (42.2%) were women. The average age when the resection was performed was 67.1±10.8, while 36 (40%) patients were younger than 65 years of age and 54 (60%) were no less than 65 years of age. 49 (54.4%) patients had cancer in right colon, whereas 41 (45.6%) in left colon. Regarding to histologic grade of colon cancer, 66 (73.3%) patients had the grade of I-II, while 24 (26.7%) patients had the grade of III-IV. As for TNM stage, 42 (46.7%) were in stage I-II and 48 (53.3%) were in stage III-IV. For tumor size, 34 (37.8%) patients had the tumor smaller than 5 cm, while 56 (62.2%) had the tumor no less than 5 cm in size. Among the 90 patients, positive lymph nodes were found in 44 (48.9%) of them, distant metastasis was observed in 5 (5.6%) and lymphovascular invasion was discovered in 5 (5.6%).

**Table 1:** Relationships between TFAM/p53 expression and clinicopathological characteristics in 90 colon adenocarcinoma cases

	Number (%)c	p53 expression (%)		*p* value	TFAM expression (%)		
		Low	High		Low	High	
Total	90(100)	37(41.1)	53(58.9)		39(43.3)	51(56.7)	
Average years	67.1±10.8	67.1±10.8	67.1±10.8		65.5±11.7	68.3±9.9	
<65	36(40.0)	16(43.2)	20(37.7)	0.665	19(48.7)	17(33.3)	0.193
≥65	54(60.0)	21(56.8)	33(62.3)		20(51.3)	34(66.7)	
Gender							
Male	52(57.8)	19(51.4)	33(62.3)	0.386	27(69.2)	25(49.0)	0.084
Female	38(42.2)	18(48.6)	20(37.7)		12(30.8)	26(51.0)	
Location							
Right colon	49(54.4)	19(51.4)	30(56.6)	0.671	22(56.4)	27(52.9)	0.832
Left colon	41(45.6)	18(48.6)	23(43.4)		17(43.6)	24(47.1)	
Histologic grade							
I~II	66(73.3)	29(78.4)	37(69.8)	0.469	30(76.9)	36(70.6)	0.632
II~ III	24(26.7)	8(21.6)	16(30.2)		9(23.1)	15(29.4)	
TNM stage							
I-II	42(46.7)	27(73.0)	15(28.3)	0.001	24(61.5)	18(35.3)	0.019
III- IV	48(53.3)	10(27.0)	38(71.7)		15(38.5)	33(64.7)	
Tumor size (cm)							
<5 cm	34(37.8)	11(29.7)	23(43.4)	0.269	13(33.3)	21(41.2)	0.514
≥5 cm	56(62.2)	26(70.3)	30(56.6)		26(66.7)	30(58.8)	
Lymphovascular Invasion	5(5.6)	1(2.7)	4(7.5)	0.645	1(2.6)	4(7.8)	0.384
Positive lymph nodes	44(48.9)	9(24.3)	35(66.0)	0.001	13(33.3)	31(60.8)	0.012
Distant metastasis	5(5.6)	2(5.4)	3(5.7)	1.000	0(0.0)	5(9.8)	0.063

### Expression of p53 and TFAM in colon adenocarcinoma tissues and corresponding paracancerous tissues

**Figure 1:**
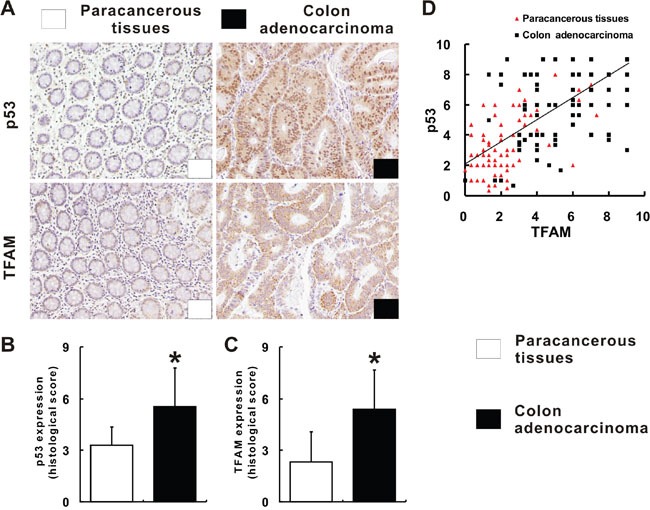
p53 and TFAM expression in colon adenocarcinoma tissues Immunohistochemistry of p53 and TFAM for colon adenocarcinoma and corresponding paracancerous tissues **A.** Scoring of p53 and TFAM staining according to staining index described in methods **B**, **C.** The linear correlation between p53 and TFAM expression in colon adenocarcinoma and corresponding paracancerous tissues **D.**

While p53 showed nuclear staining, TFAM staining was found to be inside of cytoplasm, in colon adenocarcinoma and paracancerous tissues (Figure 1A). Judging from the histological score, it was found that p53 expression was significantly higher in colon adenocarcinoma tissues than in paracancerous tissues (*p* = 0.001; Figure 1B). TFAM expression was also observed to be significantly higher in colon adenocarcinoma tissues than in paracancerous tissues (*p* = 0.01; Figure 1C). Considering the p53 and TFAM expression together, co-expression of p53 and TFAM was found with positive linear correlation (r = 0.598, *p* = 0.001; Figure 1D). Moreover, it was revealed that high co-expression of p53 and TFAM was more frequent in colon adenocarcinoma tissues than to paracancerous tissues (Figure 1D).

### Correlation of p53 and TFAM expression with progression to metastasis in human colon adenocarcinoma

In order to investigate the correlation of p53 as well as TFAM and clinicopathological characteristics, respectively, the patients were divided into two groups according to staining of either p53 or TFAM in colon adenocarcinoma tissues (Table 1). For p53 staining, two subgroups were divided according to high or low p53 expression (Table 1). Of all 90 cases, 37 (41.1%) were revealed as low p53 expression and 53 (58.9%) were found to be high p53 expression. A significant correlation between p53 expression and TNM stage as well as positive lymph nodes was observed (*p* = 0.001 and *p* = 0.001, respectively). Regarding to TNM stage I-II, percentage of patients with low p53 expression prevailed over patients with high p53 expression (73.0% *vs*. 28.3%). In contrast, in the context of TNM stage III-IV, 71.7% of patients had high p53 expression while 27% of patients had low p53 expression. Besides, it was likely that patients with high p53 expression had higher risk of developing positive lymph nodes compared with patients who had low p53 expression (66.0% *vs*. 24.3%, *p* = 0.001). No other significant correlation was found between p53 expression and other clinicopathological factors, including the association between p53 expression and metastasis of colon cancer, which might be totally uncorrelated (*p* = 1.000).

Similarly, two subgroups were divided according to the level of TFAM expression (Table 1). For all of 90 cases, 39 (43.3%) were identified as low TFAM expression and 51 (56.7%) were found to be high TFAM expression. Association between TFAM expression and TNM stage as well as positive lymph nodes was found to be significant (*p* = 0.019 and *p* = 0.012, respectively). As for TNM stage I-II, percentage of patients with low TFAM expression was more than patients with high TFAM expression (61.5% *vs*. 35.3%). However, for TNM stage III-IV, 64.7% of patients had high TFAM expression while 38.5% of patients had low TFAM expression. In addition, patients with high TFAM expression had higher risk of developing positive lymph nodes than patients with low TFAM expression (60.8% *vs*. 33.3%, *p* = 0.012). No other significant correlation was identified between TFAM expression and other clinicopathological factors, including the association between TFAM expression and metastasis of colon cancer (*p* <0.05).

### Correlation of p53 and TFAM expression with 5-years survival rate

**Figure 2:**
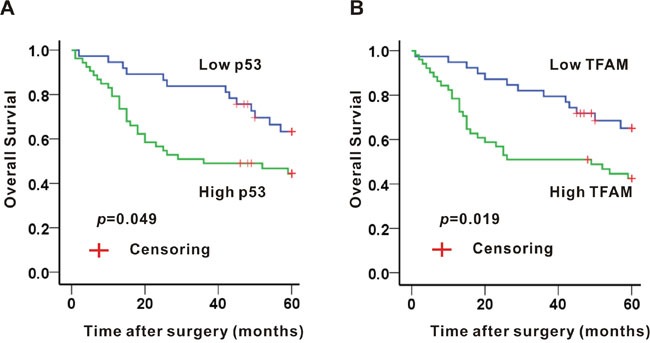
Association of p53 **A.** or TFAM **B.** expression and 5-year survival in colon adenocarcinoma tissues.

The overall 5-year survival of 90 patients was 53.3%. The median 5-year survival was 36 months (range from 1 to 60 months). Based on the expression of either p53 or TFAM, Kaplan-Meier method was administered to estimate the survival rate (Figure 2).

A significant difference was established between p53 expression and 5-year survival rate (*p* = 0.049; Figure 2A). The overall survival with high p53 expression at 5 years was 45.3%, whereas the overall survival with low p53 expression was 64.9%. Median 5-year survival rate of high and low p53 expression was 36 months (range from 1 to 60 months) and 60 months (range from 2 to 60 months), respectively.

With regard to TFAM expression, high TFAM expression was related to a low 5-year survival rate (*p* = 0.019; Figure 2B). 43.1% of patients with high TFAM expression survived at 5 years, while 66.7% of patients with low TFAM expression survived at the same time point. Median 5-years survival rate of high and low TFAM expression was 48 months (range from 1 to 60 months) and 60 months (range from 1 to 60 months), respectively.

### Factors correlated with overall survival

We further performed multivariate analysis by Cox regression to estimate the factors affecting overall survival (Table 2). Among the factors identified, we noticed that advanced TNM stage, large tumor burden, presence of distant metastasis, and high TFAM expression were significantly related to poor overall survival (*p* = 0.041, *p* = 0.029, *p* = 0.028, *p* = 0.042, respectively). Worth mentioning, p53 expression level was not found to be an independent prognostic factor predicting the overall survival (*p* = 0.349).

**Table 2:** Multivariable cox regression analysis of overall survival in 90 colon adenocarcinoma cases

Features	HR	(95% CI)	*p* value
Average years (<65 *vs*. ≥65)	0.611	0.271-1.381	0.237
Gender (Male *vs*. Female)	1.816	0.855-3.726	0.104
Location (Left *vs*. Right)	1.400	0.712-2.754	0.329
Histologic grade (I(II vs. II(III)	0.858	0.380-1.934	0.711
TNM stage (I-II vs. III- IV)	0.458	0.116-0.924	0.041
Tumor size (<5 cm vs. ≥5 cm)	0.420	0.193-0.913	0.029
Lymphovascular Invasion (No vs. Yes)	0.501	0.148-1.695	0.266
Positive lymph nodes (No vs. Yes)	0.740	0.144-3.793	0.718
Distant metastasis (No *vs*. Yes)	0.246	0.071-0.857	0.028
TFAM expression (Low *vs*. High)	0.421	0.287-1.014	0.042
p53 expression (Low *vs*. High)	0.677	0.300-1.532	0.349

HR: hazard ratio; CI: confidence interval.

### p53 and TFAM expression in colorectal cancer cell lines

**Figure 3:**
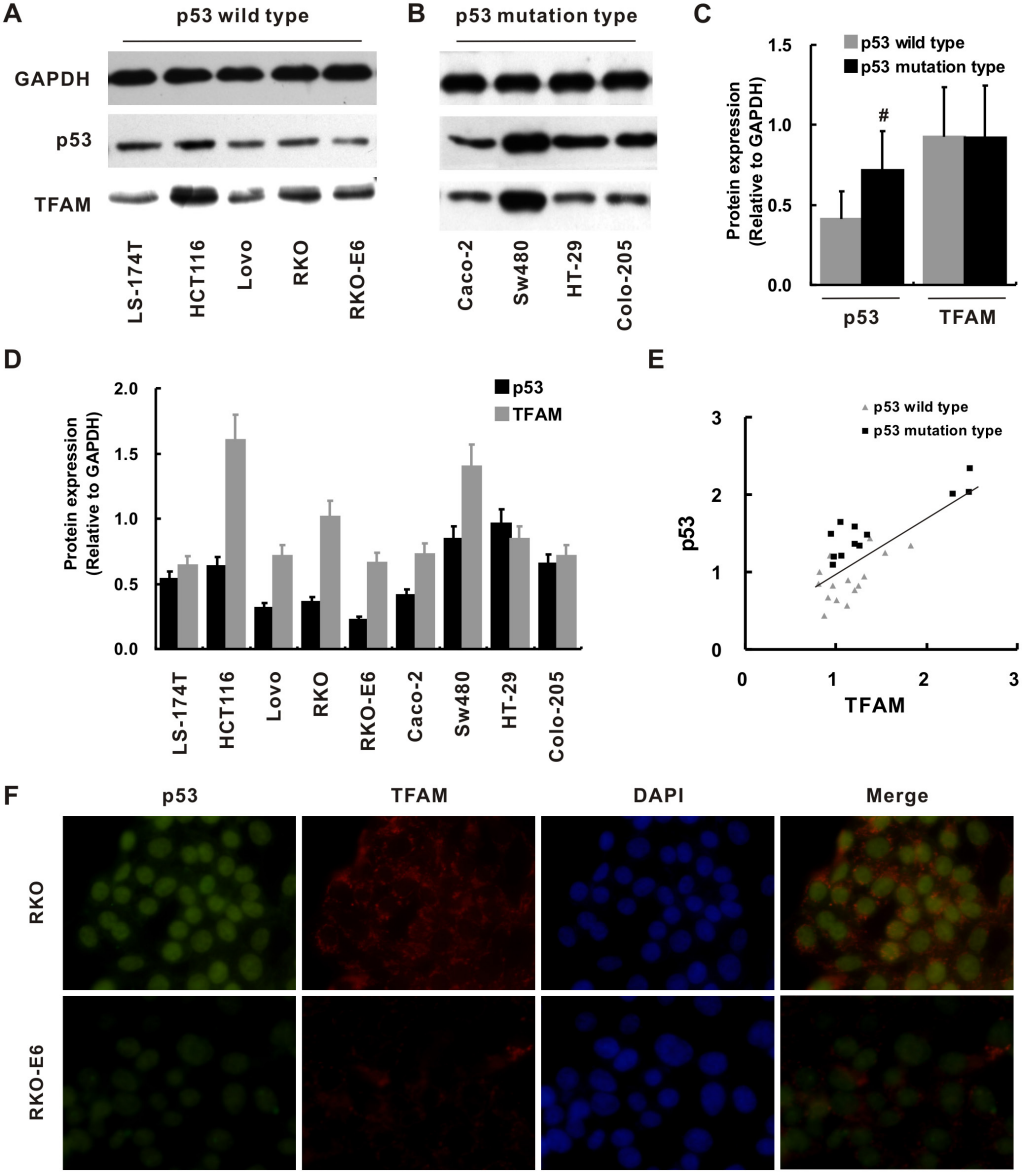
p53 and TFAM expression in different colorectal cancer cell lines Western blot for determination of p53 and TFAM expression in cell lines of p53 wild type **A.** and p53 mutation type **B.** Quantitative analysis of p53 and TFAM expression comparing p53 wild type with p53 mutation type **C.** Quantitative analysis of p53 and TFAM expression in individual cell lines **D.** The linear correlation between p53 and TFAM expression in p53 wild type and p53 mutation type cell lines **E.** Immunofluorescent staining of p53, TFAM and DAPI in RKO and RKO-E6 cell lines **F.**

Then, we examined the p53 and TFAM expression in colorectal cancer cell lines and the relationship between p53 and TFAM expression to each other. p53 and TFAM expression of colorectal cancer cell lines LS-174T, HCT116, Lovo, RKO, RKO-E6, Caco-2, Sw480, HT-29 and Colo-205 was determined by Western blot (Figure 3A-3D). Quantification of protein levels was normalized to GADPH. p53 expression was higher in the cell lines of p53 mutation type compared with that of p53 wild type (*p* < 0.05), while overall TFAM expression was not apparently different between them (Figure 3C). Then, we quantitatively investigated the p53 and TFAM expressions of individual cells (Figure 3D). A relatively high TFAM expression could be observed in cells with high p53 expression (HCT116 and SW480). Furthermore, a positive linear correlation was found to confirm the relationship between p53 expression and corresponding TFAM expression (r = 0.753, *p* = 0.001; Figure 3E).

Due to its p53 loss, RKO-E6 cell line has weaker p53 staining compared to RKO (Figure 3A), which was confirmed quantitatively by analyzing p53 protein expression (*p* = 0.023; Figure 3D). Intriguingly, TFAM expression of RKO-E6 was also significantly lower compared with that of RKO (*p* = 0.015).

### Up-regulation of TFAM expression by p53

**Figure 4:**
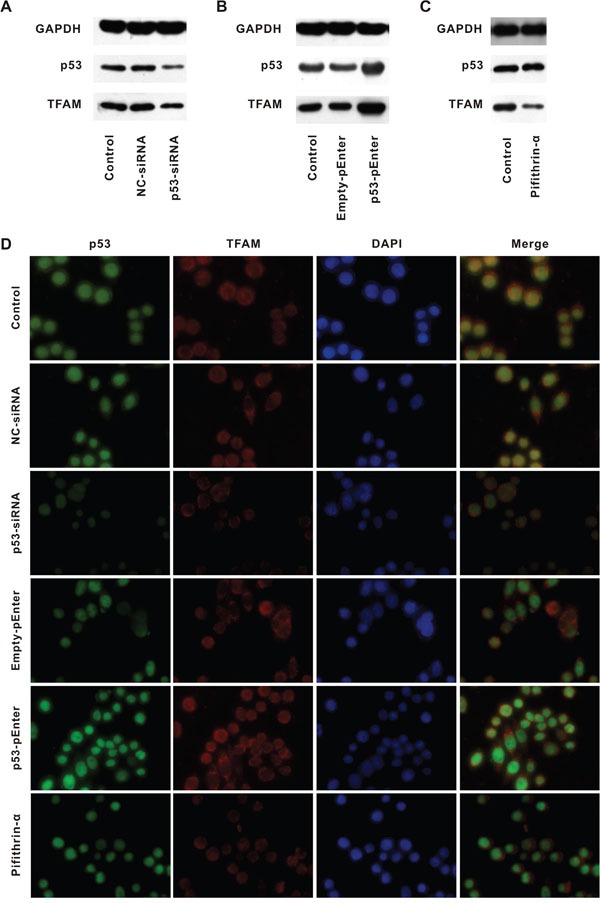
TFAM expression following alteration of p53 expression Western blot for determination of p53 and TFAM expression in cell lines treated with NC- or p53-siRNA **A.** Empty- or p53-pEnter **B.** and pifithrin-α **C.** compared with control. Immunofluorescent staining of p53, TFAM and DAPI in cell lines with different treatment, compared with control **D.**

We further investigated whether it was p53 that upregulated TFAM (Figure 4).

HCT116 was used as control and p53 as well as negative control siRNAs oligo was transfected into HCT116 (p53-siRNA and NC-siRNA, respectively, Figure 4A). Transfection of p53-siRNA was aimed to suppress p53 expression. HCT116 with p53-siRNA had a lower p53 as well as TFAM expression compared with that of control and HCT116 with NC-siRNA. Then, HCT116 was transfected with p53-pEnter and Empty-pEnter (Figure 4B). p53-pEnter could lead to p53 overexpression. HCT116 with p53-pEnter had a higher p53 as well as TFAM expression than that of control and HCT116 with Empty-pEnter. Besides, HCT116 was treated with Pifithrin-α, a p53 inhibitor (Figure 4C). Compared with control, TFAM expression of HCT116 with Pifithrin-α treatment was low, while p53 expression was not affected. These results were confirmed by immunofluorescent staining (Figure 4D). In general, in every circumstance, low TFAM expression was associated with low p53 expression (or inhibition of p53), and *vice versa*.

### TFAM do not regulate p53

**Figure 5:**
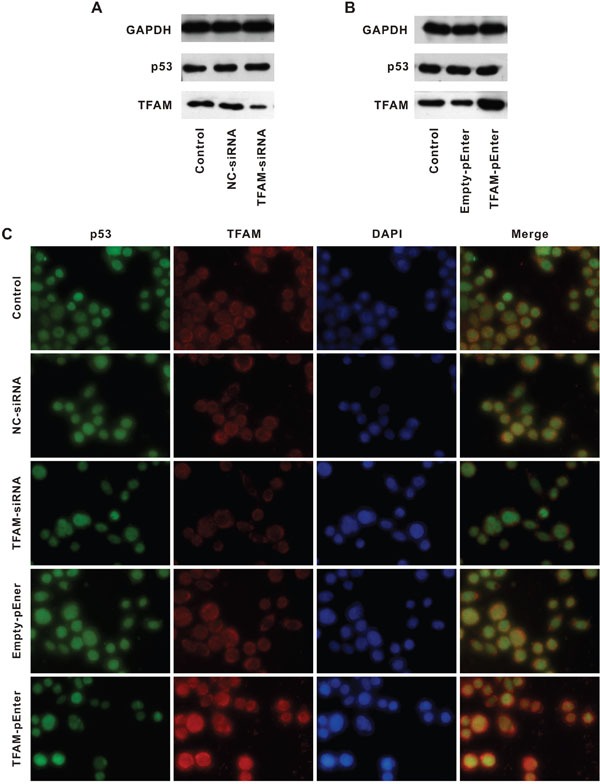
p53 expression following alteration of TFAM expression Western blot for determination of p53 and TFAM expression in cell lines treated with NC- or TFAM-siRNA **A.** and Empty- or TFAM-pEnter **B.** compared with control. Immunofluorescent staining of p53, TFAM and DAPI in cell lines with different treatment, compared with control **C.**

In addition, we also investigated whether TFAM could upregulate p53 (Figure 5).

TFAM and negative control siRNAs oligo was transfected into HCT116 (TFAM-siRNA and NC-siRNA, respectively, Figure 5A). The purpose of transfection with TFAM-siRNA was to suppress TFAM expression. HCT116 with TFAM-siRNA had a lower TFAM but normal p53 expression compared with that of control and HCT116 with NC-siRNA. Then, HCT116 was transfected with TFAM-pEnter and Empty-pEnter (Figure 5B). TFAM-pEnter could result in TFAM overexpression. HCT116 with TFAM-pEnter had a higher TFAM expression than that of control and HCT116 with Empty-pEnter, though p53 expression was not altered. The results were confirmed by immunofluorescent staining (Figure 5C). Thus, p53 expression remained unchanged even if TFAM expression was low or high.

### p53 enhance TFAM expression via binding to TFAM promoter

**Figure 6:**
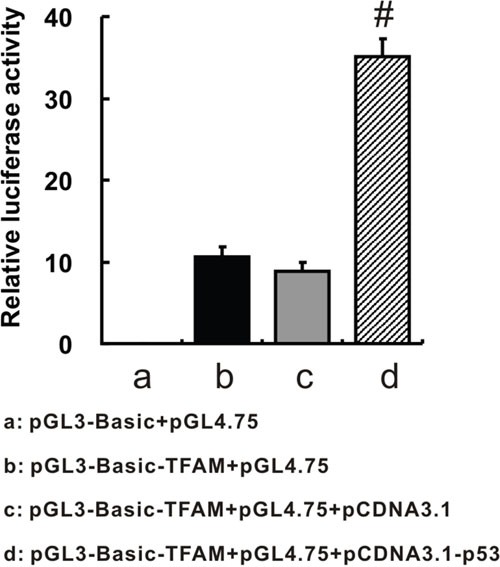
TFAM promoter region-driven luciferase expression Relative luciferase activities were shown after different transfection patterns. pGL3-Basic: a firefly luciferase reporter vector; pGL4.75: a Renilla luciferase vector, working as an internal control; pGL3-Basic-TFAM: pGL3-Basic construct with TFAM promoter region (−1486 to +185 of TFAM gene); pCDNA3.1-p53: a plasmid which could highly express p53 after transfection.

To validate whether p53 interacts with TFAMpromoter region, we performed dual-luciferase reporter assay (Figure 6). Significant increment of relative luciferase activity by 4-fold was induced by elevated p53 expression due to pCDNA3.1-p53 transfection (35.039 ± 2.303; *p* = 0.001), compared with pCDNA3.1 transfection (8.820 ± 1.166). Therefore, it indicated that p53 could enhance TFAM expression via binding to TFAMpromoter.

### Increase of mitochondrial copy number by p53 and TFAM

**Figure 7:**
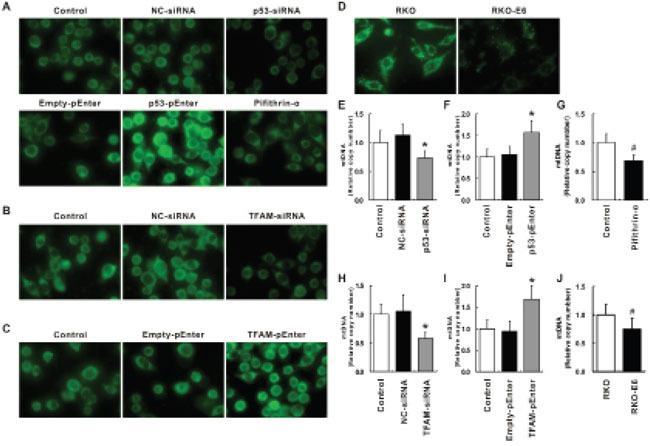
Immunofluorescent staining of mitochondria and relative mtDNA copy number Immunofluorescent staining of mitochondria for cells treated with NC-, p53- or TFAM-siRNA, Empty-, p53- or TFAM-pEnter and pifithrin-α **A**, **B**, **C.** as well as RKO and RKO-E6 **D.** Relative mtDNA copy number of cells treated with NC-, p53- or TFAM-siRNA, Empty-, p53- or TFAM-pEnter and pifithrin-α expressed as percentage of control **E**, **F**, **G**, **H**, **I.** as well as RKO-E6 expressed as percentage of RKO **J.**

HCT116 treated with the abovementioned siRNAs oligo and plasmid was further analyzed for mitochondrial morphology and mtDNA copy number (Figure 7). Mitochondria were visualized by using fluorescent probe Mito-Tracker Green. It could be found under fluorescence microscope that, when p53 was inhibited by p53-siRNA or Pifithrin-α, less mitochondria with lower staining intensity could be identified; when p53 was induced by p53-pEnter, more mitochondria with higher staining intensity could be identified (Figure 7A). In support of the observation, mtDNA copy number, determined by qRT-PCR, was found to be lower in cells treated with p53-siRNA or Pifithrin-α (*p* < 0.05; Figure 7E, 7G) while mtDNA copy number was higher in cells treated with p53-pEnter (*p* < 0.05; Figure 7F). The mitochondrial morphology and mtDNA copy number of control was comparable with cells treated with NC-siRNA and Empty-pEnter.

As for TFAM, when TFAM was inhibited by TFAM-siRNA, less mitochondria with lower staining intensity could be identified (Figure 7B); when TFAM was induced by TFAM-pEnter, more mitochondria with higher staining intensity could be identified (Figure 7C). Further investigation of mtDNA copy number in these scenario supported the observation: mtDNA copy number was found to be lower in cells treated with TFAM-siRNA (*p* < 0.05; Figure 7H) while mtDNA copy number was higher in cells treated with TFAM-pEnter (*p* < 0.05; Figure 7I). These results suggest mtDNA copy number is related to p53 as well as TFAM expression.

Furthermore, analysis of mitochondrial morphology as well as mtDNA copy number of RKO and RKO-E6 was performed (Figure 7D, 7J). Under fluorescence microscope, more mitochondria with higher staining intensity could be observed in RKO than that in RKO-E6 (Figure 7D). In addition, more mtDNA copy number was detected in RKO than in RKO-E6 (*p* < 0.5; Figure 7J). Considering the fact that RKO-E6 has a lower p53 expression, the TFAM expression should also be lower in RKO-E6 according to the results stated previously, resulting in a lower mtDNA copy number in RKO-E6 than in RKO, which indicates a relationship among p53, TFAM and mtDNA copy number.

## DISCUSSION

mtDNA copy number changes in the context of cancer [3]. However, controversial results have been reported concerning the relationship of mtDNA copy number with colorectal cancer. Some suggested there is a decrease in mtDNA copy number [18–20], while others observed a mixed change or even an increase of mtDNA copy number in patients with colorectal cancer [4, 21–23]. However, the variable results found in these researches might be the reflection of different mechanisms lying behind the regulation of mtDNA copy number. It was reported that both p53 [24] and TFAM [25] have an influence on mtDNA copy number, but in colorectal cancer, the relationship among p53, TFAM and mtDNA copy number is unknown. In the present study, we have revealed that co-expression of p53 and TFAM was more common in colon adenocarcinoma tissues than in paracancerous tissues of patients with colon cancer. Patients with either high p53 or high TFAM expression were prone to developing advanced TNM stage and positive lymph nodes, and having a low 5-year survival rate. However, after multivariate survival analysis, p53 expression was not associated with survival rate while consistently, advanced TNM stage, large tumor burden, presence of distant metastasis, and high TFAM expression were correlated with poor overall survival. Then, after investigating the relationship between p53 and TFAM expression *in vitro*, we found that p53 could upregulate TFAM but not the other way around. Additionally, it was also observed that either p53 or TFAM expression could induce an elevation of mtDNA copy number. Taken together, it could be speculated that p53 regulates TFAM expression and alteration of mtDNA copy number succeeds to the change of either p53 or TFAM expression. To our knowledge, there is currently no research exploring the relationship among p53, TFAM and mtDNA copy number all together in colorectal cancer. Thus, this study could provide further insights into understanding colorectal cancer.

p53, known as the guardian of genome [26], has a critical role of inducing apoptosis and preventing oncogenesis [27]. Accumulation of p53 protein in cancer is common [13], which does not have to relate to p53 mutation in colorectal cancer [14, 15]. Consistent with the literacy [13], we found that colon adenocarcinoma tissue from our patients had high p53 expression. In addition, advanced TNM stage and positive lymph nodes, and a low 5-year survival rate were related to p53 expression. Different from our observation, some authors suggested that, for all patients or patients with a body mass index higher than 30 kg/m^2^, no correlation could be observed between p53 expression and survival rate [28]. The difference between our results might be mainly ascribed to different methods involved for evaluating level of p53 expression, plus we did not record the body mass index of our patients. Additionally, we did not observe a relationship between p53 expression and overall survival. This might be explained simply as a result of the role of p53 as an upstream factor, and the key factor affects survival should be its downstream effector. For example, in a recent study, high p53 expression was not associated with poor survival in pancreatic cancer whereas its postulated downstream factor RAB27B was associated with prognosis [29]. In another research, p53 expression did not show prognostic value either, but similarly, cyclin A, a potential target of p53, was observed to be the prognosticator of cervical carcinoma [30]. Specifically, in our study, the downstream factor was TFAM, which functioned directly to influence prognosis. Finally, regarding to p53 mutation, we observed that cells from p53 mutation type did not show an absolute overexpression of p53 protein (Figure 3D). Thus, we validated that p53 mutation did not necessarily guarantee an overexpression of p53 protein [15].

TFAM, packing whole mtDNA, is essential to maintenance and transcription of mtDNA [31], whose mutation is also found to be associated to some colorectal cancer [32]. In our study, high TFAM expression was revealed in the TMA of patients with colon adenocarcinoma, which was correlated to advanced TNM stage, high incidence of positive lymph nodes, a low 5-year survival rate and poor prognosis. There is a limited number of research available discussing the relationship between TFAM expression and the outcome of colorectal cancer. According to Nakayama *et al* [9], TFAM positivity indicates a worse survival rate with higher rate of lymph node metastasis and advanced TNM staging in colorectal cancer; however, they also demonstrated that distant metastasis is also related to positive TFAM expression, which was not seen in our study. Actually, we did observe a tendency of higher incidence of distant metastasis in patients with higher TFAM expression but the result was not significant. This might be attributed to more patients involved in their study as well as the fact that we only recruited patients with colon adenocarcinoma but they also included patients with rectal cancer. Nevertheless, more research should be conducted to find out the relationship of TFAM expression with distant metastasis.

It is already established that p53 interactis with TFAM to regulate mtDNA content [33–35]. However, in colorectal cancer, no research has reported the effect of p53 on TFAM. In addition, it's not clear whether TFAM could regulate p53. According to our observation, in different colorectal cancer cell lines, a positive linear correlation was established between the level of p53 expression and the level of TFAM expression. Besides, by inducing p53 expression, TFAM expression was also enhanced while the suppression of p53 could lead to the change of TFAM expression in the same direction. On the contrary, neither overexpression nor inhibition of TFAM could alter p53 expression. Thus, we confirmed that p53 could regulate TFAM expression [33], and we also suggested that TFAM could not do the opposite, indicating p53 being an upstream regulator of TFAM. We further used dual-luciferase reporter assay to determine how p53 affects TFAM expression. From our observation, it is safe to say that p53 promotes TFAM expression by interacting with TFAM promoter. According to a previous study [36], after an acute bout of treadmill exercise in the mouse, p53 could translocate to nucleus and it is speculated that TFAM expression is upregulated because of the interaction between nuclear DNA and p53 [16]. Thus, these results were consistent with ours and we proved that p53 could enhance TFAM expression via TFAM promoter.

As previously stated [18–23], mixed results have been published on the change of mtDNA copy number in colorectal cancer, suggesting the complexity of regulation on mtDNA copy number. According to other researchers, mtDNA copy number could be influenced via many aspects other than p53 or TFAM, such as proliferator-activated receptor gamma coactivator alpha (PGC-1α) [5], nuclear respiratory factor 2 (NRF-2) [6], polymerase γ gene POLG (POLG) [7] and so on. However, it is only recently demonstrated that p53 could form a complex with TFAM, which could further bind at the D-loop region of mtDNA, regulating the transcription activity of mtDNA [35]. Thus, it will be interesting to find out more about the relationship among p53, TFAM and mtDNA. Our finding was that the change of either p53 or TFAM expression could result in the alteration of mtDNA copy number. In general, high p53 or TFAM expression could lead to the increased mtDNA copy number, while with low p53 or TFAM expression, decrease of mtDNA copy number occurred. Considering p53 as an upstream molecule regulating TFAM, we deduce that p53 might, at least to some degree, alter mtDNA copy number via its regulation on TFAM. This conclusion is supported by our results that both TFAM and mtDNA copy number changed in the same direction with p53 expression. Further investigation, such as inducing p53 and inhibiting TFAM simultaneously and quantitatively to observe the change of mtDNA copy number (since it is reported that the mtDNA copy number is associated with the total TFAM protein level in a manner of direct proportion [37]), should be conducted to warrant our thought.

As a summary, we have found a correlation of both p53 and TFAM expression to advanced TNM stage, positive lymph nodes and low 5-year survival rate in patients with colon adenocarcinoma. Also, TNM stage, tumor size, distant metastasis, and TFAM expression but not p53 expression were identified as independent prognostic factors. Besides, p53 could regulate TFAM expression as an upstream regulator via TFAM promoter, and both of them could influence mtDNA copy number in colorectal cell lines. Thus, we speculate p53 might regulate mtDNA copy number via its regulation on TFAM expression. Our study highlighted the relationship among p53, TFAM and mtDNA copy number, which provides novel insights into understanding colorectal cancer and innovative treatment might be developed based on this.

## MATERIALS AND METHODS

### Tissue microarray (TMA)

Colon adenocarcinoma TMA utilized in the study was provided by National Engineering Center for Biochip at Shanghai (Shanghai, China). 90 patients who were diagnosed with colon cancer and received surgical treatment between April 2008 and November 2008 were included in the TMA samples. The detailed clinical and pathologic information of these patients was shown in Table 1. All patients were followed up from the date of surgery until September 2014.

### Immunohistochemistry (IHC) and scoring

TMA sections were deparaffinized in xylene and a series of ethanol dilutions. Antigen retrieval was carried out by heating sections in 10 mM sodium citrate buffer to 98°C for 15 min. Endogenous peroxidase activity was blocked with treatment of 3% H_2_O_2_. Then, the section incubation was performed with p53 antibody (1:100, Santa Cruz Biotechnology, Santa Cruz, CA, USA) and TFAM antibody (1:100, Abcam, Cambridge, UK) overnight at 4°C, for assessment of p53 and TFAM, respectively. Subsequently, it was followed by 30 min of incubation with horseradish peroxidase-conjugated secondary antibody kits (ZSGB Bio, Beijing, China) at 4°C for 30 min. Finally, sections were stained with 3, 3′-diaminobenzidine tetrahydrochloride solution after which the counterstain with haematoxylin was done. Each section was assessed by three independent pathologists with blinded information, clinically and pathologically. The expression of p53 and TFAM in sections was scored semi-quantitatively by positive percentage and intensity of stained preparation (staining index = positive × intensity score). The positively staining percentage of tumor cells was scored as: 0, no staining; 1, <20%; 2, 20-75%; 3, >75%. The staining intensity of tumor cells was graded as follows: 0, negative; 1, weak; 2, moderate; 3, strong staining. Based on the abovementioned staining index, a final score of 0-4 was thought to be low expression of p53 and TFAM, while a final score of 4.1-9 was deemed as high expression.

### Cell culture and treatments

Human colon adenocarcinoma cell LS-174T, HCT116, Lovo, RKO, RKO-E6, Caco-2, Sw480, HT-29 and Colo-205 was obtained from the Type Culture Collection of the Chinese Academy of Sciences (Shanghai, China). RKO, RKO-E6 and Caco-2 were cultured with minimum essential medium (MEM, HyClone, Logan, UT, USA) containing essential amino acids (Sigma, St. Louis, MO, USA) and 10% fetal bovine serum (FBS, Gibco, Logan, UT, USA). LS-174T and HT-29 cells were cultured with Dulbecco's modified Eagle's medium (DMEM, HyClone) containing 10% FBS. HCT116, Lovo, Sw480 and Colo-205 cells were performed with PRMI-1640 (HyClone) containing 10% FBS. Cells were incubated in a humidified atmosphere of 37°C, with 5% CO_2_. Prior to the treatment of pifithrin-α for 24 h, cells were serum-starved with culture medium containing 2% FBS for 6 h. Pifithrin-α (10 mmol/L) was dissolved in dimethyl sulphoxide (DMSO) immediately before incubation. The final concentration Pifithrin-α (Sigma) and DMSO was 10 μM and 0.1% in each treated cells, respectively.

### Gene silencing of p53 and TFAM by small interfering RNA (siRNA)

The siRNAs oligo (p53, TFAM and negative control) was synthesized by Transheep Bio (Shanghai, China). siRNA was transfected into 70% confluent HCT116 cells with Lipo2000 (Invitrogen, Carlsbad, CA, USA) The procedure of siRNA transfection was followed with the instruction of manufacturer. p53-siRNA: GCAUCUUAUCCGAGUGGAATT, UUCCA CUCGGAUAAGAUGCTT. TFAM-siRNA: GACGAAAC UCGUUAUCAUATT, UAUGAUAACGAGUUUCGU CTT. Negative control (NC-siRNA): UUCUCCGAACGUG UCACGUTT, ACGUGACACGUUCGGAGAATT. At 24 h and 72 h after transfection, cells were harvested and analyzed by quantitative real-time PCR (qRT-PCR) and Western blot, respectively, to confirm the silence efficacy of p53 or TFAM.

### Over-expression of p53 and TFAM by plasmid transfection

The open reading frame (ORF) of p53 in pEnter (p53-pEnter), ORF of TFAM in pEnter (TFAM-pEnter) and empty vector plasmid pEnter (Empty-pEnter, Transheep Bio, Shanghai, China) were transfected into HCT116 cells by using LipoFiter (Shanghai Hanheng, Shanghai, China) according to the manufacturer's protocol. In short, HCT116 cells which were in logarithmic growth phase were seeded on 6 cm dishes with the density of 5×10^5^ cells per dish. p53-pEnter, TFAM-pEnter or empty-pEnter DNA (3 μg) and LipoFilter (20 μL) were added to each well when HCT116 cells grew to around 50-70% confluence. Then, after 6 h of transfection, serum-free medium was replaced with fresh PRMI-1640 and cultured for another 72 h after which subsequent experiments were performed. p53 and TFAM protein overexpression were both verified by qRT-PCR and Western blot.

### Western blot analysis for p53 and TFAM protein expression

Extraction of whole proteins was performed for cultured cells with protein extraction kit (Nanjing Kaiji, Nanjing, China). Same amount of protein (30 μg) from each sample were transferred respectively to PVDF membrane (Millipore, Billerica, MA, USA). PVDF membranes were treated with 5% non-fat dry milk dissolved in incubation buffer to block non-specific binding sites. Then, PVDF membranes were incubated with primary antibodies directed against GAPDH (1:5000, Abcam), TFAM (1:2000) and p53 (1:2000) in primary antibody dilution buffer at 4°C overnight. After washing, membranes were incubated with appropriate horseradish-peroxidase-conjugated secondary antibodies (1:10000, Santa Cruz) for 2 h at 37°C. The bands were then visualized with ECL detection kit (Nanjing Kaiji), determined by Quantity One software 4.5.0 (Bio-Rad, Hercules, CA, USA) and normalized to GAPDH.

### Immunocytofluorescence staining for p53 and TFAM

HCT116 cell culture was performed on coverslips, at the bottom of 24-well plates. The cells were fixed with 4% paraformaldehyde prior to permeabilization with 0.1% Triton X-100. After blocking with 5% goat serum, incubation was performed with primary antibodies against p53 and TFAM overnight at 4°C followed by incubation with each corresponding TRITC-conjugated and FITC-conjugated secondary antibodies. Cell nuclei were stained with 4′, 6-diamino-2-phenylindole (DAPI, Roche, Basel, Switzerland). Slides were coverslipped with anti-fading medium and observed under a fluorescence microscope (CX41, Olympus, Tokyo, Japan).

### Mito-tracker green

The fluorescent probe Mito-Tracker Green (Beyotime, Jiangsu, China) was used to detect mitochondria of cultured cells. Cells were cultured on coverslips at the bottom of 24-well plates. At the end of corresponding treatments, cells were incubated with 50 nM Mito-Tracker Green in RPMI-1640 for 30min at 37°C in darkness. After washing, fluorescent staining was observed under a fluorescence microscope.

### Quantitative real-time PCR (qRT-PCR) for mtDNA copy number

Extraction of mitochondrial DNA (mtDNA) was performed with QIAamp DNA Mini kit (Qiagen, Hilden, Germany) and real time PCR was done by using mtDNA specific primer (forward primer 5′-TACTCACCAGACGCCTCAACCG-3′ reverse primer 5′-TTATCGGAATGGGAGGTGATTC-3′), and the other primer pair for β-actin gene was run in parallel to standardize the input DNA (forward primer 5′-CGGGAAATCGTGCGTGACAT-3′, reverse primer 5′-GAAGGAAGGCTGGAAGAGTG-3′). Primers were synthesized by Invitrogen. Reactions were carried out in a 25 μL volume containing 12.5 μl 2×SYBR Premix Ex Taq (BioRad), 1.0 μL forward primer (10mM), 1.0 μL reverse primer (10 mM), 2.0 μL DNA template (100 ng) and 8.5 μL dH_2_O. The cycling conditions were as follows: 95°C for 30 sec followed by 40 cycles of 95°C degree for 5 sec and 60°C for 30 sec. Final results were presented as percentage of mtDNA copy number relative to that of control (in the case of comparison between RKO and RKO-E6, the result was the percentage of mtDNA copy number relative to that of RKO).

### Dual-luciferase reporter assay

Dual-luciferase reporter assay was utilized to confirm whether p53 could directly upregulate TFAM expression. HEK293 cells were cultured with DMEM containing 10% FBS. Transfection was done with FuGENE 6 Transfection Regent (Promega, Madison, WI, USA) according manufacturer's instruction after cells were grown to 70%-80% confluence. A TFAM responsive luciferase construct which encodes the firefly luciferase reporter gene controlled by TFAM promoter region was transfected (pGL3-Basic-TFAM). The TFAM promoter region was a fragment of human TFAM gene promoter, generated by amplification of a region from −1486 to +185 of TFAM gene. By the way, pGL3-Basic is a vacant construct without TFAM promoter region. pGL4.75 is a Renilla luciferase vector, working as an internal control. pCDNA3.1-p53 is the plasmid which could highly express p53 after transfection. Cells were harvested 30 hours after transfection, underwent lysis, and were measured for luminescence with Dual-Luciferase Reporter Assay System (Promega, Madison, WI, USA). Finally, Firefly- Luc/Renilla Luc ratio was recorded. The results were expressed as relative luciferase activity, after normalized to the Renilla luciferase internal control.

### Statistical analysis

All data were expressed as mean ± SD. SPSS 19.0 software (SPSS, Chicago, IL, USA) was utilized for analysis. Quantitative data were analyzed by one-way ANOVA followed with SNK multiple comparison test. Analysis of the difference of clinicopathological parameters between low and high p53 and TFAM expression groups, respectively, was conducted by χ^2^ test. Survival rates as well as survival curves were derived from the Kaplan-Meier method and the results were compared by log-rank test. The correlation between p53 and TFAM expression was assessed by the Pearson Correlation analysis. Multivariable Cox proportional hazards regression model was applied to determine hazard ratios of overall survival. A *p*-value of < 0.05 was considered significant.
